# Ethanol Extract of *Antrodia camphorata* Grown on Germinated Brown Rice Suppresses Inflammatory Responses in Mice with Acute DSS-Induced Colitis

**DOI:** 10.1155/2013/914524

**Published:** 2013-05-30

**Authors:** Dong Ki Park, Hye-Jin Park

**Affiliations:** ^1^Cell Activation Research Institute, Konkuk University, 1 Hwayang-dong, Gwangjin-gu, Seoul 143-701, Republic of Korea; ^2^Department of Bioscience and Biotechnology, Konkuk University, Gwangjin-Gu, Achasan-rho 263, Seoul 143-701, Republic of Korea

## Abstract

The anti-inflammatory activity of *Antrodia camphorata* (AC) grown on germinated brown rice (CBR) extract was evaluated *in vitro* and *in vivo*. CBR suppressed the release of nitric oxide (NO) and prostaglandin (PG) E2 from lipopolysaccharide-(LPS-)stimulated RAW264.7 cells. CBR inhibited the level of inducible nitric oxide synthase (iNOS) and cyclooxygenase-(COX-)2 proteins, and it activated p38-MAPK, extracellular signal-related kinases (ERK), and NF-**κ**B in LPS-stimulated RAW264.7 macrophages. LPS-induced tumor necrosis factor-**α** (TNF-**α**) and interleukin-6 (IL-6) mRNA expression was reduced in CBR-treated RAW264.7 cells. In concert with *in vitro* data, CBR suppressed the levels of dextran-sulfate-sodium-(DSS-)induced iNOS and COX-2 proteins in the colon tissue. CBR treatment inhibited activated p38-MAPK, ERK, and NF-**κ**B proteins in the colon tissue of DSS-induced mice. TNF-**α** and IL-6 mRNA expression was reduced in DSS+CBR-treated mice. The disease activity index and histological scores were significantly lower in CBR-treated mice (500 mg/kg/day) than in DSS-treated mice (*P* < 0.05 versus DSS). This is the first report of anti-inflammatory activity of CBR in DSS-induced acute colitis. These results suggest that CBR is a promising, potential agent for preventing acute colitis through the inhibition of NF-**κ**B signaling and its upstream signaling molecules, including MAPKs.

## 1. Introduction

Inflammatory bowel diseases (IBDs), including ulcerative colitis (UC) and Crohn's disease, are immunologically mediated chronic intestinal disorders. The incidence of IBDs increased considerably over the past half century in developed countries. The rapidity of these developments may be the result of exposure to nongenetic factors (as opposed to genetic factors) introduced through changes in the diet and lifestyle of genetically susceptible individuals, which can cause aberrant immune responses that lead to IBD. The modality of these diseases is commonly accompanied by severe intestinal inflammation, including abdominal pain, diarrhea, rectal bleeding, and weight loss [1–3]. Prolonged UC and chronic UC further develop into colorectal cancer at a high rate. In spite of substantial progress for treating UC, definitive therapies, which do not result in an eventual relapse, are not available to date, and limiting drug-induced toxicity is a huge challenge [4]. The abnormal mucosal immune and inflammatory responses in IBD are often characterized by induced production of proinflammatory cytokines, reactive oxygen, and nitrogen species and the activation of macrophages. An increase in the nuclear factor kappa B (NF-*κ*B) signaling molecules has been detected in the mucosa of patients with IBD and in a murine colitis model. Inhibition of NF-*κ*B activity, with a specific p65 antisense oligonucleotide, is effective in preventing experimental models of IBD, and it markedly downregulates proinflammatory cytokine production in intestinal macrophages in IBD patients [5, 6]. This activated signaling leads to the production of proinflammatory cytokines (e.g., tumor necrosis factor-alpha (TNF-*α*), interleukin-1*β* (IL-1*β*), IL-6, IL-8, and IL-12) and the expression of inflammatory-related proteins (e.g., cyclooxygenase-2 (COX-2) and inducible nitric oxide synthase (iNOS)). Therefore, the overexpression of proinflammatory cytokines and inflammatory-related proteins contributes to the inflammatory cascade in the pathological progression of colitis [7]. 

Recently, natural products and their derived compounds have been proposed as novel drug candidates owing to their broad spectra of therapeutic effects with low toxicity. *Antrodia camphorata* (AC) is an indigenous and rare mushroom of the Polyporaceae family that grows only on the inner heart rot of the native Taiwanese tree *Cinnamomum kanehirai* Hay (Lauraceae). AC is used in traditional Chinese medicine for treating food and drug poisoning, diarrhea, abdominal pain, hypertension, pruritis (skin itch), and liver dysfunction [1, 8–10]. Recent studies have highlighted various biological functions of AC, including antioxidant, anti-inflammatory, antihepatitis, antitumor, and immunomodulatory properties [1, 11, 12]. However, obtaining massive amounts of AC in nature is not easy because it is a parasitic fungus that only grows in the inner cavity of *Cinnamomum kanehirai* Hay (Lauraceae; Bull camphor tree) at an altitude of 200–2000 m in Taiwan. To solve the low production yield of AC, our study group developed novel culture methods by inoculating AC mycelia on germinated brown rice (CBR) that are rich in nutrients and biologically active compounds. Recently, our group identified various anticancer activities of CBR against B16F10 cancer cells [13]. Despite the reported beneficial effects of AC, its pharmacological actions against inflammatory bowel disease have not been documented. In this study, we explored the ability of CBR to regulate inflammatory responses and the underlying mechanism by using macrophage-mediated inflammatory conditions and DSS-induced acute colitis.

## 2. Materials and Methods

### 2.1. Preparation of CBR Extract

*A. camphorata* grown on germinated brown rice (CBR) was provided by Cell Activation Research Institute (CARI, Seoul, Republic of Korea). Germinated brown rice was used as culture media for *A. camphorata *mycelia. *A. camphorata *mycelia was inoculated onto the germinated brown rice and cultured for 8 weeks at 20–25°C. Dried 8-week CBR was ground into a fine powder using a grinder. Powder (2 kg) was extracted with 80% ethanol (EtOH) at 20–25°C. After filtration, the ethanol extracts were dried with a rotary evaporator under vacuum and the extract was stored at −20°C. Authenticated voucher specimens of CBR (Kucari 1101) are deposited in the Herbarium at College of Bioscience and Biotechnology, Konkuk University, Seoul, Republic of Korea.

### 2.2. Cell Culture

RAW 264.7 macrophages were purchased from the American Type Culture Collection (ATCC; Rockville, MD, USA) and cultured in regular media consisting of Dulbecco's modified Eagle's medium (DMEM; Invitrogen, Carlsbad, CA, USA), 10% fetal bovine serum (FBS; Invitrogen), and 100 U/mL penicillin-streptomycin (Sigma). 

### 2.3. NO, *PGE*_2_, and TNF-*α* Assays

Nitrite concentrations in RAW 264.7 cell culture media were measured using the Griess reaction as described previously [14]. PGE_2_ levels were measured using enzyme-linked immunosorbent kits (Cayman Chemical Co., Ann Arbor, MI, USA) as described previously [15]. Enzyme-linked immunosorbent assay (ELISA) was performed according to the manufacturer's protocol for measuring the level of TNF-**α** (Pierce Biotechnology, Rockford, IL, USA). For these assays, cells (5  ×  10^4^ cells/mL) were pretreated in the presence or absence of CBR or AC extract for 1 h before lipopolysaccharide (LPS; 1 *μ*g/mL, Sigma) stimulation for 20 hours. 

### 2.4. Isolation of Nuclear Extracts

RAW264.7 macrophages were pretreated in the presence or absence of CBR extract (0, 50, and 100 *μ*g/mL) for 1 h before stimulation with 1 *μ*g/mL LPS. Nuclei were isolated and nuclear proteins were extracted using the Nuclear Extraction Kit from Panomics (Panomics Inc., Redwood City, CA, USA).

### 2.5. Western Blotting

Cell extracts and colon tissues were separated on a 10% SDS-PAGE gel, transferred to a sheet of nitrocellulose membrane (Millipore; Billerica, MA, USA), and blocked in 5% nonfat milk for 1 h. Samples were probed with the following primary antibodies: iNOS, COX-2, NF-*κ*B, phospho-p38, p38, phosphoextracellular signal-related kinases (ERK1/2), ERK1/2, and *β*-actin (all from Santa Cruz, CA, USA). The secondary antibodies used were horseradish peroxidase-conjugated goat antirabbit or anti-mouse antibodies (Pierce, Rockford, IL, USA). An enhanced chemiluminescence reaction was performed using a SuperSignal West Femto Enhancer Kit (Pierce), and the positive bands were detected on an X-ray film.

### 2.6. Quantitative Real-Time Polymerase Chain Reaction

RAW 264.7 macrophages were treated with CBR extract (0, 50, and 100 *μ*g/mL) followed by LPS stimulation. Total RNA was isolated using an RNA isolation kit according to the manufacturer's instructions (Toyobo, Japan) and then reverse-transcribed. Thereafter, RT-PCR was performed using an ABI7500 thermal cycler (Applied Biosystems, Foster City, CA, USA) with a set of primers corresponding to iNOS (QT00100275), TNF-*α* (QT00104006), and GAPDH (QT01658692) (all from Qiagen, Valencia, CA, USA). Relative iNOS and TNF-*α* mRNA levels were normalized with GAPDH and calculated using the 2^ΔΔ^Ct method.

### 2.7. Animal Experiment

C57BL/6 mice (6 weeks old, female) were purchased from the DaeHan Experimental Animal Center (Eumsung, Korea). Mice were acclimatized under controlled, specific pathogen-free (SPF) conditions for 1 week prior to the experiment. All mice were housed in individual cages and fed standard laboratory chow in an animal room with 12 h light/dark cycles. All animals were handled following the guidelines of the Institutional Animal Care and Use Committee (IACUC) at Konkuk University (Seoul, Republic of Korea). The authorization code number from IACUC was ku11069. 

### 2.8. Experimental Protocol

Acute colitis was induced in C57BL6/N mice by adding DSS (MP Biologicals, USA) to drinking tap water (3.5% v/v) for 9 days, as previously described [1, 3, 16]. Mice were randomly assigned to 4 groups (*n* > 5 per group). The groups were as follows: Group 1, normal mice administered with drinking water and vehicle for 11 days; Group 2, mice pretreated with a vehicle for 2 days and then coadministered with a vehicle and 3.5% DSS for 9 days; Group 3, mice pretreated with CBR for 2 days and then coadministered with 3.5% DSS and CBR EtOH extract (200 mg/kg/day) for 9 days; and Group 4, mice pretreated with CBR for 2 days and then coadministered with 3.5% DSS and CBR EtOH extract (500 mg/kg/day) for 9 days.

### 2.9. Evaluation of the Disease Activity Index (DAI)

The DAI was used for evaluating the grade and extent of intestinal inflammation [3]. Body weight, stool consistency, and blood in the stool were monitored daily for determination of the DAI. Each score was defined as follows: for body weight loss, 0 = none, 1 = 1–5%, 2 = 6%–10%, 3 = 11%–20%, and 4 = >20%; for diarrhea, 0 = normal, 2 = loose stools, and 4 = watery diarrhea; and for blood, 0 = normal, 2 = slight bleeding, and 4 = gross bleeding. The DAI score ranged from 0 to 12 (total score) (Table 1).

### 2.10. H&E Staining and Assessment of Histological Score

Colon tissue sections were stained with hematoxylin and eosin (H&E) as previously described [3]. Stained sections were examined by light microscopy (Nikon Co., Japan) (magnifications: 100x and 200x). The histological scoring system was used for evaluating the degree of colitis with H&E images following a previously published scoring system [3]. The scoring system was as follows: 0 = no change from normal tissue; 1 = low level of inflammation with scattered infiltrating mononuclear cells (1-2 foci); 2 = moderate inflammation with multiple foci; 3 = a high level of inflammation with increased vascular density and marked wall thickening; and 4 = severe inflammation with transmural leukocyte infiltration and a loss of goblet cells.

### 2.11. Immunohistochemistry

The distal colon was dissected and a longitudinal section (1.5 cm from the anal verge) was prepared. The immunohistochemical staining of the colon section (4 *μ*m) was performed using the ImmunoCruz system (Santa Cruz, CA, USA). Tissue sections were incubated with antibodies against NF-*κ*B p65 (Santa Cruz; 1 : 50). Sections were developed by using the DAB chromogen kit (Vector laboratories, Burlingame, CA, USA) and counterstained with 1% methyl green for 1 min. The sections were observed by microscopy (Nikon Co., Japan).

### 2.12. Statistical Analysis

Data were expressed as means ± standard error of the mean. A one-way ANOVA was used for assessing significance between the control group and sample treated groups. Two-way repeated-measure ANOVAs were used to analyze the data at different time points. Statistical analysis was performed using SPSS, version 12 (SPSS Inc., Chicago, IL, USA). 

## 3. Results

### 3.1. CBR EtOH Extract Inhibited NO and PGE_2_ Production and Proinflammatory Cytokine mRNA Expression in LPS-Stimulated RAW264.7 Cells

**Figure 1:**
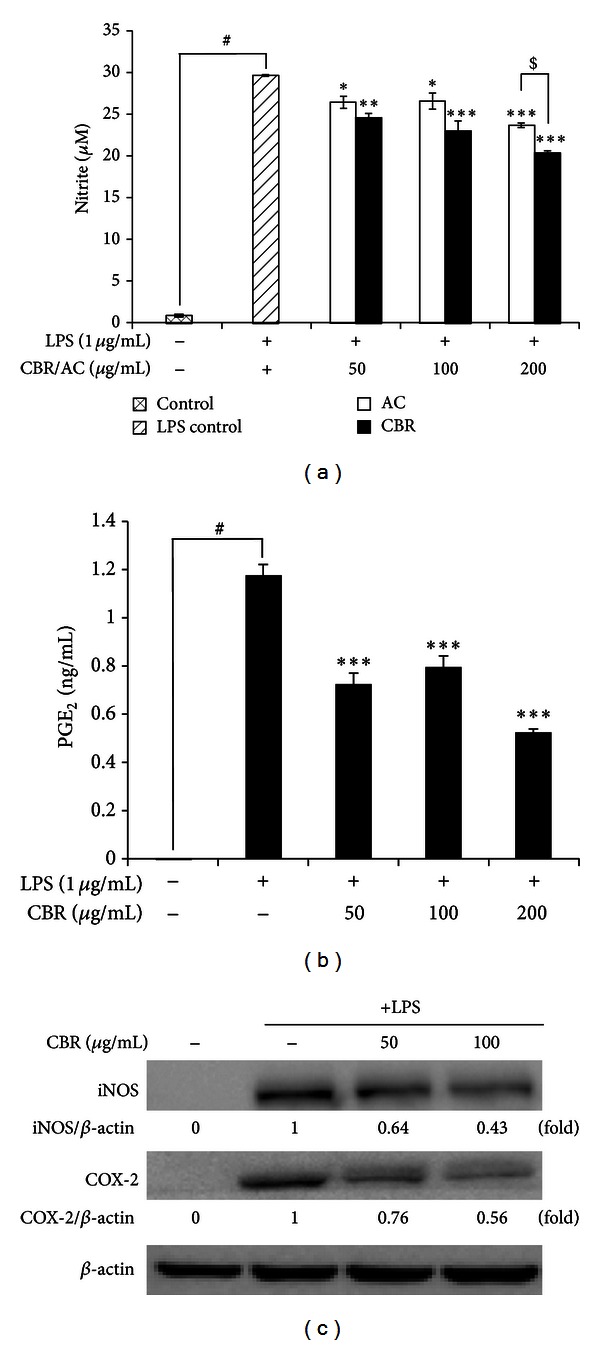
Effect of the CBR extract on NO and PGE_2_ production_,_ and iNOS and COX-2 protein expression levels in LPS-stimulated RAW264.7 macrophages. Cells were incubated for 20 h with 1 *μ*g/mL of LPS in the absence or presence of the CBR or AC extract. The CBR extract was added 1 h prior to incubation with LPS. (a, b) Nitrite and PGE_2_ production in the medium was determined using the Griess reagent and PGE_2_ EIA kit. (c) Protein expression of iNOS and COX-2 was analyzed by western blot analysis and quantified by densitometric analysis. Data show the mean ± standard error (S.E.) of 3 independent experiments. A one-way ANOVA was used for comparisons of multiple group means followed by Dunnett's *t*-test (^#^ *P* < 0.001 versus control; *P < 0.01, **P < 0.05, and ***P < 0.001 versus LPS-stimulated control; ^$^ *P* < 0.05 versus AC).

In an effort to investigate the anti-inflammatory activity of *A. camphorata *grown on germinated brown rice (CBR) and *A. camphorata* (AC), we first confirmed whether they inhibit NO production in activated macrophages. LPS (1 *μ*g/mL) was used as a positive control for macrophage activation (Figure 1(a)). CBR significantly inhibited NO production compared to AC at 200 *μ*g/mL (*P* < 0.01 versus AC). The 50% inhibitory concentrations (IC50) of NO production by CBR and AC were 312.02 ± 16.67 *μ*g/mL and 525.20 ± 15.4 *μ*g/mL, respectively (Figure 1(a)). 

**Figure 2:**
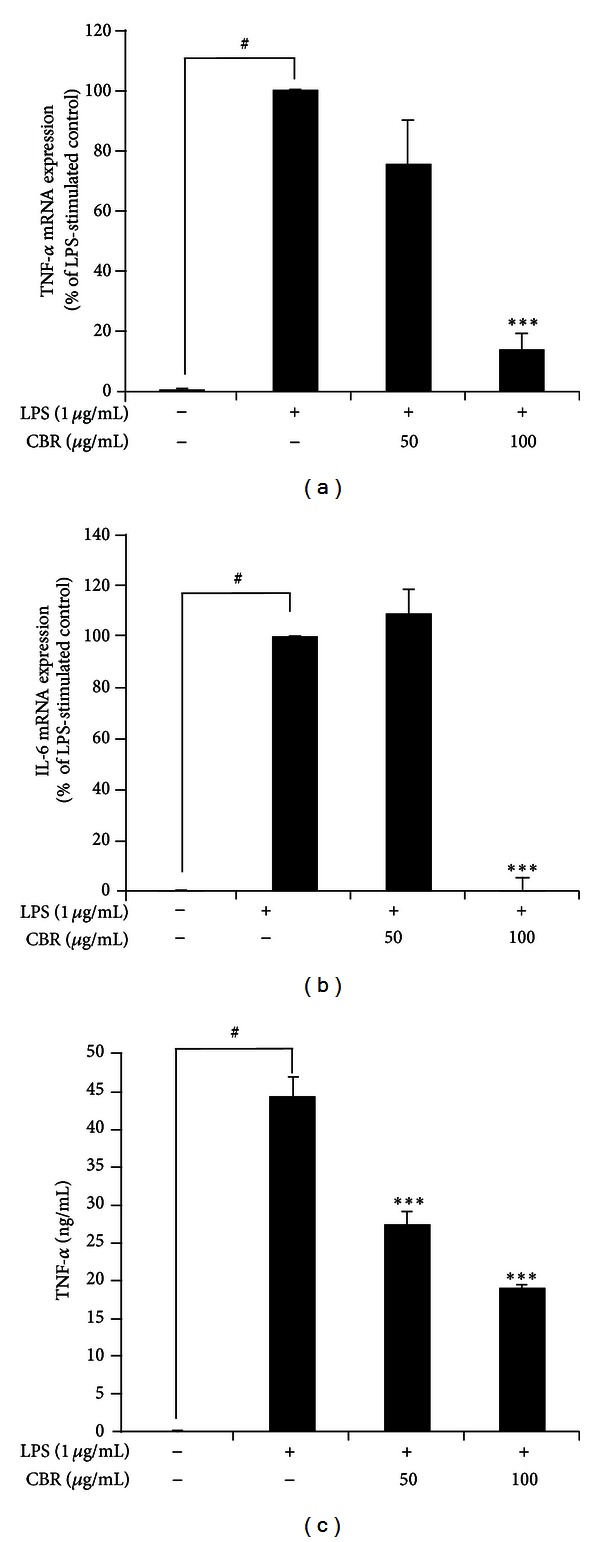
Effect of CBR extract on the level of proinflammatory cytokines in LPS-stimulated RAW264.7 macrophages. (a, b) Cells were incubated for 5 h with 1 *μ*g/mL of LPS in the absence or presence of the CBR extract. The CBR extract was added 1 h prior to incubation with LPS. Levels of TNF-*α* and IL-6 mRNA in LPS-stimulated RAW264.7 cells were analyzed by real-time polymerase chain reaction (RT-PCR) and determined by quantitative ΔΔC_T_ RT-PCR by using GAPDH mRNA as the internal control. Data represent the mean ± S.E. of 3 independent experiments. A one-way ANOVA was used for comparisons of multiple group means followed by Dunnett's *t-*test (^#^ *P* < 0.001 versus control; *P < 0.01, **P < 0.05, and ***P < 0.001 versus LPS-stimulated control).

Therefore, we chose CBR EtOH extract as a test sample for this study. CBR treatment blocked the production of PGE_2_ in LPS-stimulated RAW264.7 cells in a dose-dependent manner (Figure 1(b)). To understand the molecular inhibitory mechanism of CBR on NO and PGE_2_ production, we evaluated iNOS and COX-2 protein expression. Protein levels of iNOS and COX-2 were significantly decreased at 100 *μ*L/mL of CBR treatment (Figure 1(c)). In addition, LPS-induced IL-6 and TNF-*α* mRNA levels were significantly inhibited by CBR treatment (100 *μ*L/mL) (Figure 2). CBR (50 and 100 *μ*g/mL) significantly reduced macrophage TNF-*α* secretion from 44.3 ± 2.58 ng/mL to 27.4 ± 1.70 ng/mL and 19.0 ± 0.35 ng/mL, respectively (***P < 0.001 versus LPS-stimulated control).

### 3.2. CBR EtOH Extract Inhibited NF-*κ*B Expression in LPS-Stimulated RAW264.7 Cells

**Figure 3:**
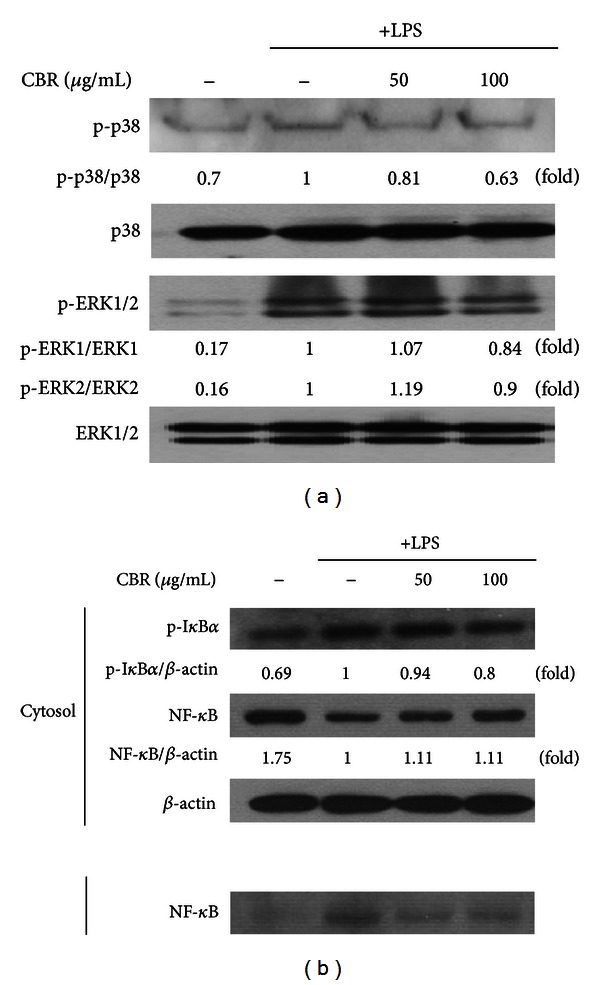
Effect of the CBR extract on MAPK and NF-*κ*B expression in LPS-stimulated RAW264.7 cells. (a, b) Cells were incubated for 30 min with 1 *μ*g/mL of LPS in the absence or presence of the CBR extract. The CBR extract was added 1 h prior to incubation with LPS. Protein expression of MAPK and NF-*κ*B was analyzed by western blot analysis and quantified by densitometric analysis.

Figure 1 shows that CBR EtOH extract inhibition could be due to the blockade of transcriptional activation induced by inflammation-regulating signaling molecules and transcription factors such as MAPK and NF-*κ*B [7]. First, we determined whether CBR inhibited phosphorylation of MAPK. CBR extract decreased the expression of phosphorylated ERK and p38-MAPK in a dose-dependent manner in LPS-induced RAW264.7 cells (Figure 3(a)), respectively. Here, we investigated whether CBR extract could suppress the activation of NF-*κ*B, using western blot analysis. NF-*κ*B activation is essential for the inflammatory response in RAW264.7 cells. Since the hyperphosphorylation of I*κ*B and its subsequent degradation is an essential step in NF-*κ*B activation by various stimuli [5]. Equivalent proteins from nucleus were used to check whether the NF-*κ*B activation was affected by CBR extract and the cytosolic proteins were used to determine the degradation of I*κ*B. We found that CBR extract strongly inhibited translocation of NF-*κ*B p65 subunit (Figure 3(b)) as well as inhibited I*κ*B*α* phosphorylation in a dose-dependent manner (Figure 3(b)). Therefore, these results strongly suggest that CBR extract can suppress the production of inflammatory mediators and proinflammatory cytokines by suppressing NF-*κ*B-mediated transcriptional activation. 

### 3.3. CBR EtOH Extract Attenuated DSS-Induced Acute Colitis Symptoms

**Figure 4:**
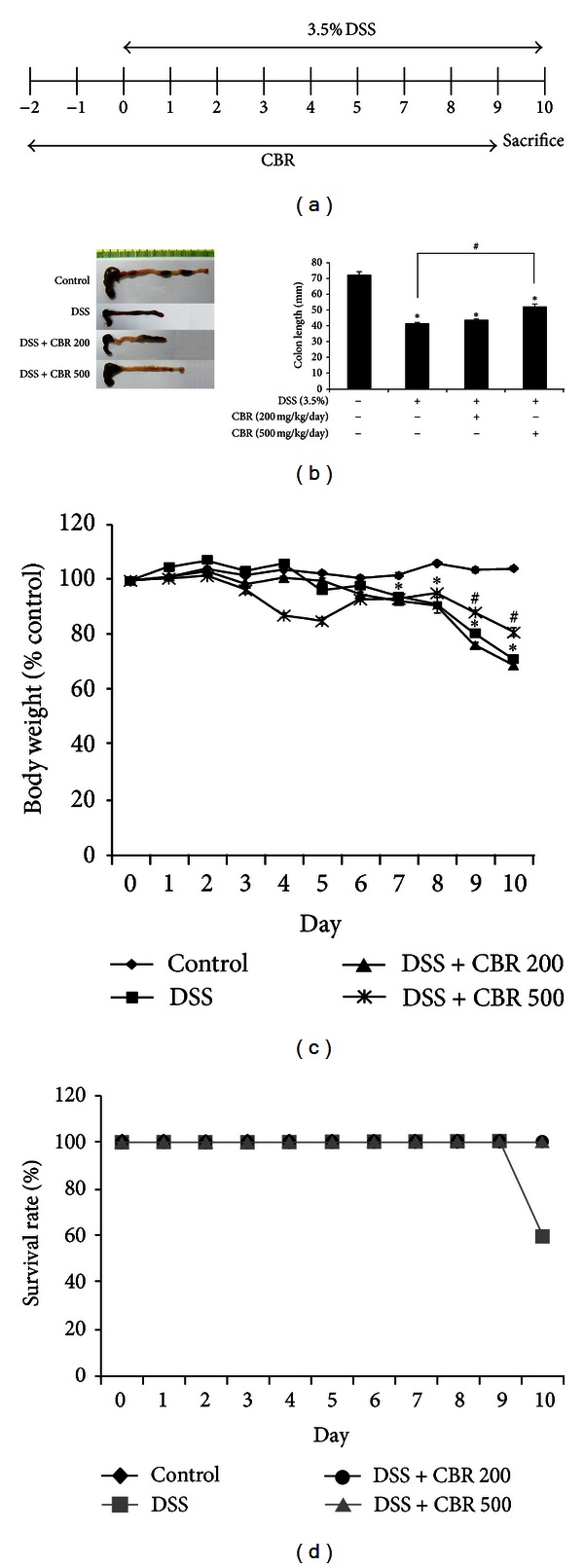
The CBR extract attenuated the severity of DSS-induced acute colitis. (a) Scheme of the experimental design. (b) Colons from each group were collected at day 10 after DSS instillation. Macroscopic images of the colons are shown (left panel). Colon length of each mouse was measured (right panel). (c) Changes in body weight were measured. Body weight was presented as the percentage of the initial weight (at day 0). Data are expressed as the mean ± S.E. (*n* = 6 per group) (*P < 0.05  versus control; ^#^ *P* < 0.05  versus DSS; two-way ANOVA followed by a post hoc analysis). (d) Difference in survival rate. This experiment was repeated twice, and similar results were obtained. DSS dextran sulfate sodium; CBR: *Antrodia camphorata* (AC) grown on germinated brown rice.

**Figure 5:**
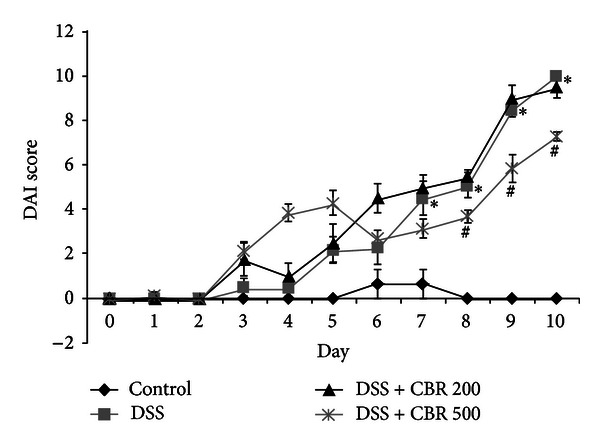
Ameliorative effects of the CBR extract on the disease activity index (DAI) score. The DAI score was monitored daily. DAI was significantly lower in CBR+3.5% DSS compared with 3.5% DSS. Data are expressed as the mean ± S.E. (*n* = 6 per group) (*P < 0.05  versus control; ^#^ *P* < 0.05  versus DSS; two-way ANOVA followed by a post hoc analysis). DSS dextran sulfate sodium; CBR AC grown on germinated brown rice.

Anti-inflammatory activities of the CBR EtOH extract were also evaluated using mice with DSS-induced acute colitis, which exhibit similar symptoms to the acute phase of human ulcerative colitis. To study the prophylactic effect of the CBR EtOH extract, mice were administered with CBR (500 mg/kg) for 2 days, followed by administration of 3.5% DSS (Figure 4(a)). It has been reported that the length of the colon is inversely linked to the severity of DSS-induced acute colitis. We found that the colon of CBR EtOH extract- (200 and 500 mg/kg/day) administered mice had been significantly longer than that of the DSS-treated group (Figure 4(b)). Changes in the body weight were checked daily during the experiment. Marked weight reductions were observed in the DSS groups, whereas the CBR EtOH extract (500 mg/kg/day) significantly attenuated DSS-induced weight reductions at 7–10 days (*P* < 0.05) (Figure 4(c)). Mortality was only observed in DSS-treated group on day 10 (Figure 4(d)). In order to determine if CBR EtOH extract can attenuate acute colitis symptoms, we quantitatively scored macroscopic clinical symptoms, using the disease activity index (DAI) (e.g., body weight loss, diarrhea, and gross bleeding) (Table 1). An induction in DAI was observed in DSS-treated mice (Figure 5). The DAI was significantly lower in CBR + DSS-treated mice compared with DSS-treated mice on days 8, 9, and 10 (*P* < 0.05). The colitis seen in mice fed CBR EtOH extract (500 mg/kg/day) was less severe and extensive (Figure 5).

### 3.4. CBR Extract Decreased Histological Changes

**Figure 6:**
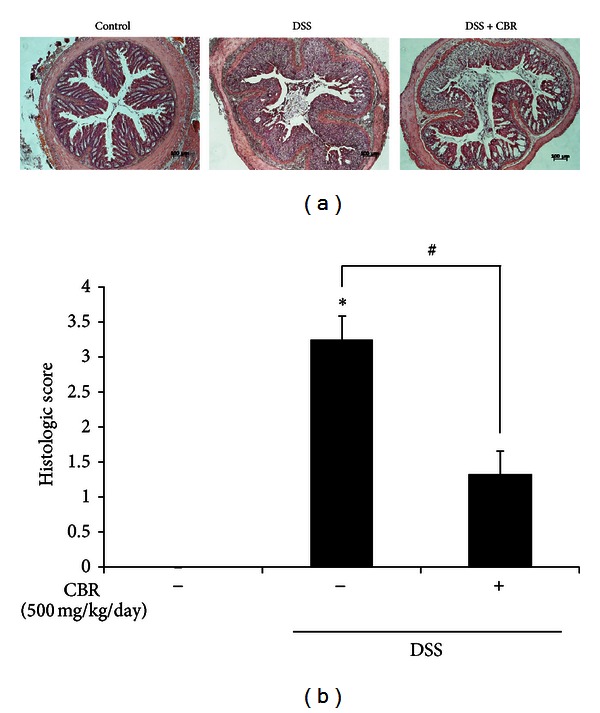
Effect of CBR extract on colonic histological changes. (a) Representative histological findings in mice with dextran-sodium-sulfate-(DSS-) induced colitis. Representative sections of colon tissues from normal mice administered with drinking water (control), mice administered with DSS-induced colitis (DSS), and mice coadministered with DSS and CBR extract (DSS+CBR). Histological changes were determined by H&E staining. Scale bar = 100 *μ*m. Original magnification: 100x. Protein expression of iNOS and COX-2 was analyzed by western blot analysis and quantified by densitometric analysis. (b) Histological score was measured. (c) Histologic score of colitis induced by DSS. Data are expressed as mean ± S.E (*n* = 6 per group). (*P < 0.05  versus control; ^#^ *P* < 0.05  versus DSS.) DSS: dextran sulfate sodium; CBR: *Antrodia camphorata *grown on germinated brown rice.

We examined the architecture of colonic structure microscopically, using hematoxylin and eosin (H&E) staining method. Mice with DSS-induced acute colitis showed edema, distorted epithelial barrier, significant loss of crypts and goblet cells, and marked infiltration of leukocytes into the mucosa and submucosa, compared to control group. CBR extract group showed relatively well-preserved mucosal and crypt structure and less infiltration of inflammatory cells in the colonic tissues compared to the DSS-treated group (Figure 6(a)). These changes paralleled differences in histological colitis scores. There histological changes were assessed by grading scores as described in Section 2. Mean histopathology scores were lower in the group fed with CBR EtOH extract (3.3 ± 0.3) than those in the DSS-treated group (1.3 ± 0.3) (Figure 6(b)). 

### 3.5. CBR EtOH Extract Treatment Attenuated iNOS, COX-2, and Proinflammatory Cytokine Expression in the Colon Tissue of Mice with DSS-Induced Colitis

**Figure 7:**
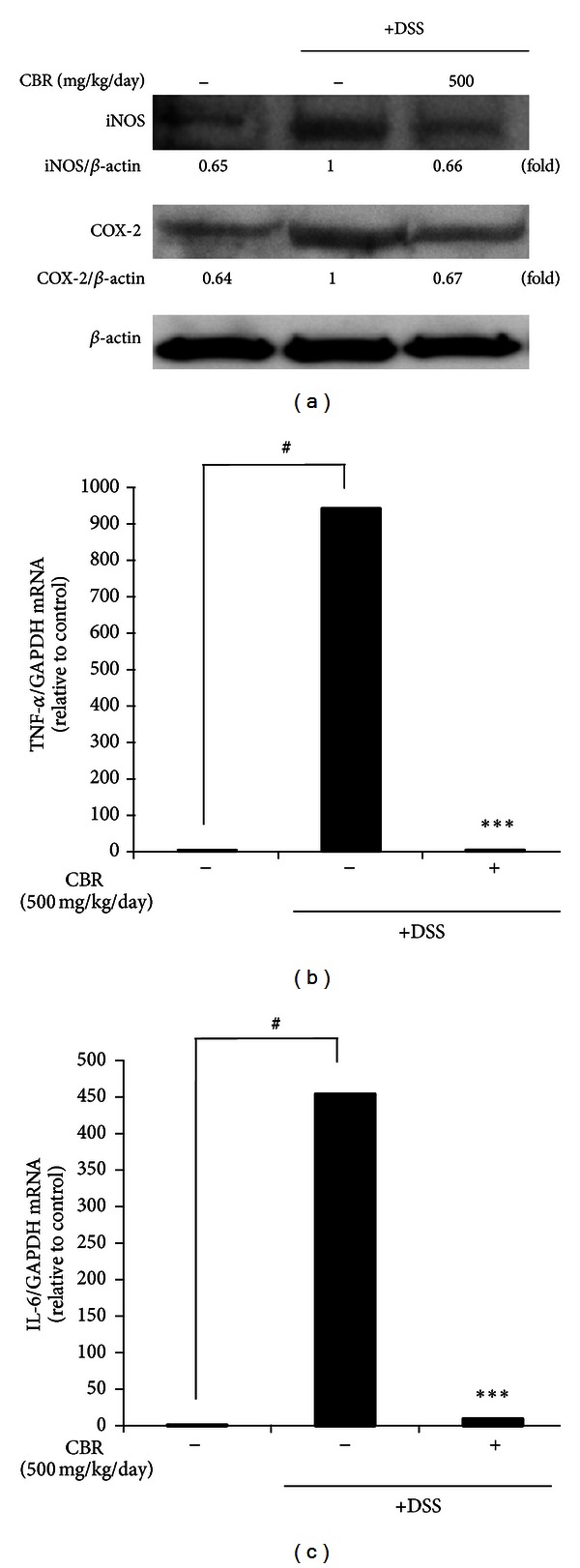
Effect of the CBR extract on the DSS-induced iNOS and COX-2 protein expression and TNF-*α* and IL-6 mRNA expression levels. (a) Immunoblotting analysis of iNOS and COX-2. Representative colon tissue lysates from normal mice administered with drinking water (control), mice administered with DSS-induced colitis (DSS), and mice coadministered with DSS and CBR extract (DSS+CBR) (left to right lane). Protein expression of iNOS and COX-2 was analyzed by western blot analysis and quantified by densitometric analysis. (b, c) TNF-*α* and IL-6 mRNA expression levels of colon tissues were analyzed by RT-PCR and determined by quantitative ΔΔC_T_ RT-PCR by using GAPDH mRNA as the internal control. Data represent the mean ± standard error (S.E.) of 3 independent experiments. A one-way ANOVA was used for comparisons of multiple group means followed by Dunnett's *t-*test (^#^ *P* < 0.05  versus control; ***P < 0.001  versus DSS).

We examined whether the inhibitory mechanism exhibited by the CBR EtOH extract could also be observed in the DSS-induced colitis model. In agreement with the *in vitro* data, reduced levels of iNOS and COX-2 proteins were observed in the CBR EtOH extract-treated group compared to the DSS-treated group (Figure 7). Colonic injury by DSS administration resulted from an increase of proinflammatory cytokine TNF-*α* [17]. It has been shown that selective blockade of TNF-*α* and IL-6 significantly decreases the severity of colitis and neutrophil/macrophage migration [18]. Next, we measured the levels of TNF-*α* and IL-6 mRNA in colonic tissue. The CBR extract significantly blocked TNF-*α* mRNA expression in the colonic tissues of mice with DSS-induced acute colitis (Figure 7).

### 3.6. CBR EtOH Extract Prevents Colonic MAPKs and NF-*κ*B Activation in the Colon Tissue after DSS Administration

**Figure 8:**
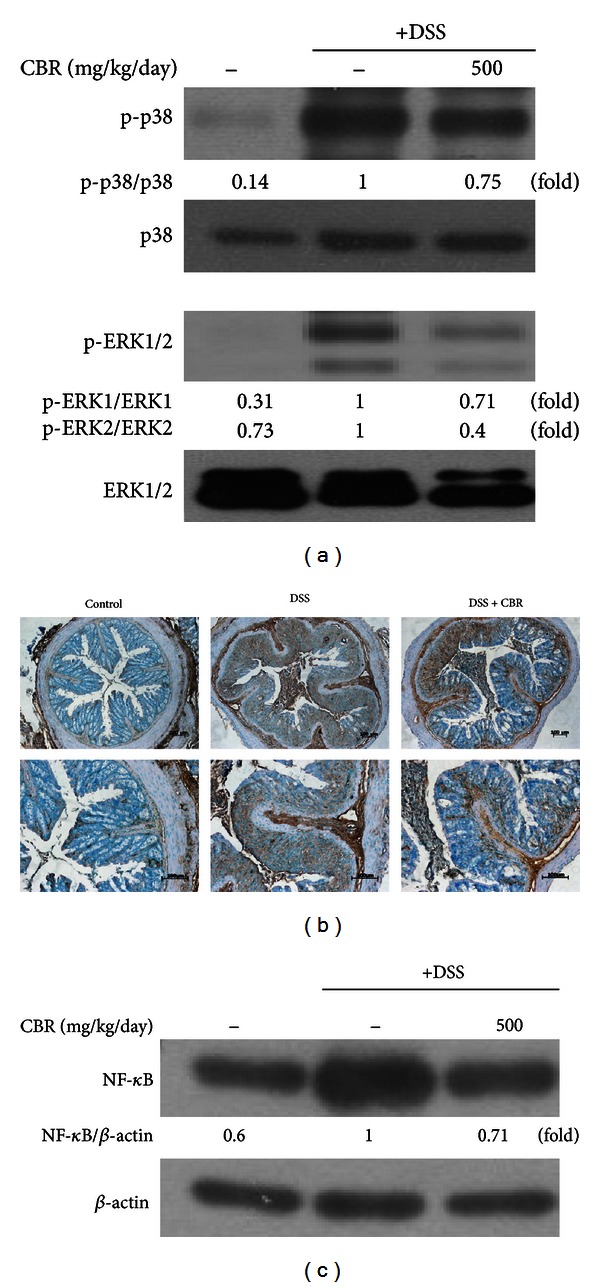
Effect of the CBR extract on the DSS-induced MAPK and NF-*κ*B protein expression. (a) Immunoblotting analysis of phosphorylated MAPKs. Representative colon tissue lysates from normal mice administered with drinking water (control), mice administered with DSS-induced colitis (DSS), and mice coadministered with DSS and CBR extract (DSS + CBR) (from left to right). Protein expression of MAPK and NF-*κ*B was analyzed by western blot analysis and quantified by densitometric analysis. (b) The immunohistochemical analysis was used to monitor the protein expression of NF-*κ*B. Scale bar = 100 *μ*m. Original magnification: 100x and 200x (top and bottom panels, resp.). (c) Immunoblotting analysis of NF-*κ*B.

Activated MAPKs and transcriptional factor NF-*κ*B primarily contribute to the major proinflammatory signaling pathways involved in colitis. Phosphorylation of ERK and p38-MAPK was upregulated in the colon tissue of experimental colitis.  Figure 3 shows that the CBR EtOH extract inhibited the phosphorylation of ERK and p38-MAPK, as well as the translocation of NF-*κ*B. Whether such inhibition by the CBR EtOH extract could also be observed in an *in vivo* disease model was assessed using a DSS-induced colitis model. DSS stimulated activation of p38 and ERK, whereas DSS-induced p38 and ERK phosphorylation diminished to 25% and 45%, respectively, in the colonic tissue after CBR extract administration (Figure 8(a)). Low levels of NF-*κ*B p65 were detected in the naive mouse colon; however, DSS induced the protein level of p65 NF-*κ*B in the colonic tissue after 7 days (Figures 8(b) and 8(c)). Immunohistochemistry results also showed that the protein expression of NF-*κ*B p65 in DSS group was distinctly elevated compared with control group (*P* < 0.01, Figure 8(c)), while the pretreatment with CBR EtOH extract (500 mg/kg, p.o.) significantly inhibited the p65 NF-*κ*B in the mouse colon tissue. Thus, this result strongly suggests that suppression of activated MAPKs (e.g., p38, ERK) and NF-*κ*B activation seems to be the key mechanism through which CBR EtOH extract modulates intestinal inflammation.

## 4. Discussion

*Antrodia camphorata* (AC) is a traditional medicine in Taiwan and China for treating liver dysfunction, food and drug poisoning, diarrhea, abdominal pain, hypertension, and inflammation [1, 8–10]. In spite of its usage as a traditional medicine, there are no papers on anti-inflammatory activities of AC against inflammatory bowel diseases (IBDs), including Crohn's disease (CD) and ulcerative colitis (UC). The aim of this study is to demonstrate anti-IBD activities of AC on germinated brown rice (CBR) and its anti-inflammatory mechanism of action.

Therefore, we first checked the anti-inflammatory effects of the CBR extract on LPS-stimulated RAW264.7 cells. LPS-stimulated macrophages release proinflammatory mediators (e.g., NO and PGE_2_) and proinflammatory cytokines such as TNF*-*α** and IL-6 [8, 19]. Overproduction of NO by macrophages provokes various inflammatory diseases [20]. CBR significantly inhibited NO and PGE_2_ production from LPS-induced macrophages with no cytotoxicity (Figure 1). The suppressive effect of CBR extract occurr at the translational level. CBR decreased the level of iNOS and COX-2 protein in LPS-induced macrophages (Figure 1(d)). CBR also suppressed mRNA expression in TNF-*α* and IL-6 (Figure 2). In order to elucidate the probable mechanisms through which the CBR extract ameliorates these inflammatory responses in experimental colitis, changes in NF-*κ*B and its upstream signaling pathway, and the relative expressions of serial signaling molecules, we performed western blot analysis. Three major groups of MAPKs include the ERK, the c-Jun NH2-terminal kinases, and p38-MAPKs, which are activated by phosphorylation. They are the upstream enzymes for NF-*κ*B. MAPK cascades propagate signals, which change gene expressions that control diverse functions (e.g., inflammatory responses in various cells like macrophages, monocytes, and epithelial cells) [21–23]. NF-*κ*B can be activated by various stimuli (e.g., proinflammatory cytokines, microbes and microbial products, and oxidative stress) that signal its activation through the catalytic I*κ*B kinase β (IKK*β*) [6]. IKK*β* phosphorylates NF-*κ*B-bound I*κ*Bs in the cytoplasm and targets their degradation, thereby leading to the subsequent release of NF-*κ*B dimmers. Subsequently, these NF-*κ*B dimmers translocate from the cytoplasm to the nucleus and activate the transcription of multiple *κ*B-dependent target genes (e.g., TNF-*α*, IL-1*α*, and IL-6), intercellular adhesion molecules, COX-2, and iNOS [24, 25]. We found that CBR suppressed ERK and p38 phosphorylation, NF-*κ*B translocation, and I-*κ*B phosphorylation in LPS-stimulated RAW264.7 cells (Figure 3).

Finally, the anti-inflammatory activity of CBR was examined using the DSS-induced colitis model. DSS is administered to mice to mimic UC symptoms by disrupting the epithelial cell barrier and activating mucosal macrophages, which, in turn, causes an inflammatory response [3]. We pretreated animals with CBR (200 and 500 mg/kg/day) for 2 days before DSS was administered. This study design may be helpful for patients in the acute phase of UC in that the prophylactic administration of CBR may be recommended.

The protective effects of the CBR extract on DSS-induced colitis were assessed by macroscopic and histological analyses. Improved characteristic symptoms of IBD (e.g., diarrhea, bloody stools, abdominal pain, and weight loss) and histopathological characteristics (e.g., crypt abscesses, crypt distortion, ulceration, and the infiltration of large numbers of neutrophils, monocytes, and lymphocytes) were observed in the CBR-fed groups (Figure 4). At 500 mg/kg/day of the CBR EtOH extract, the severity of DSS-induced acute colitis symptoms was markedly reduced. This was evidenced by a decrease in weight loss, DAI score, and histopathological scores (*P* < 0.05; Figures 5 and 6). According to the interspecies dosage scaling calculation, the human equivalent dose of the CBR EtOH fraction in our *in vivo *study was approximately 427.26 mg for an adult weighing 60 kg; this dose is applicable in future clinical investigations.

Oxidative stress is a major cause of tissue damage and inflammation [1]. Sustained, high levels of NO production, especially when mediated by iNOS in the colon, play a role in the pathology of IBD [26–28]. Several synthetic iNOS inhibitors were effective in suppressing DSS-induced colitis symptoms in mice [26–28]. Marked elevated levels of PGE_2_ and COX-2 were also observed during inflammation, including DSS-induced colonic injury [29]. Our data demonstrated that CBR extracts decreased the levels of iNOS and COX-2 in the colonic tissues of DSS-administered mice. This finding suggests that the CBR extract acts as an iNOS and COX-2 inhibitor, thus protecting the colon from DSS-induced tissue injury and inflammation by reducing nitrosative stress. It was reported that high levels of proinflammatory cytokines, including TNF-*α* and IL-6, were detected in DSS-administered mice. TNF-*α* damages the epithelial barrier of the colon and induces apoptotic death of epithelial cells in the colon [1]. Consistent with the findings from a previous study, the CBR extract significantly attenuated the levels of TNF-*α* and IL-6 mRNA expression in the colonic tissues of mice with DSS-induced acute colitis (Figure 7).

Recent experiments showed the importance of p38 in UC, where the use of p38 and ERK inhibitors abrogated colitis [30, 31]. Moreover, a recent study demonstrated that it can be effective for treating human IBD. p38 is known to regulate several target genes that ultimately control infiltration of monocytic cells, acute intestinal inflammation, intestinal electrolyte and water secretion, and cytokine production. Furthermore, it upregulates COX-2 expression in intestinal epithelial cells [30]. Growing evidence indicated that the inhibition of NF-*κ*B activity (e.g., a direct blockade of RelA (p65), suppression of I*κ*B*α* degradation, or IKK*β* activity) may alleviate the severity of intestinal inflammation [5]. Numerous natural anti-inflammatory products (e.g., *Cissus quadrangularis*, *Taraxacum officinale*, and *Sanguisorba officinalis*) [32, 33] have been reported as NF-*κ*B inhibitors, which can be regarded as potential anti-inflammatory drug candidates. Indeed, many recent studies have focused on developing novel anti-inflammatory drugs targeted to regulate NF-*κ*B activity [34]. In agreement with the *in vitro* study, the CBR extract inhibited phosphorylation of p38 and ERK1/2 as well as the protein level of the NF-*κ*B p65 subunit (Figure 8). Therefore, CBR may possess anti-inflammatory activities through the modulation of the NF-*κ*B signaling pathway and its upstream signaling enzymes, which could be a primary target for the treatment of IBD. Total flavonoid content in the CBR extract was determined to be 0.18%, as determined by a previously published method [35] (data not shown). Further studies will explore the effects of compounds derived from the CBR extract on the acute colitis model.

## 5. Conclusion

 In summary, this study showed, for the first time, that the CBR extract significantly reduces DSS-induced colitis symptoms through the prevention of body weight loss and colon shortness and decreasing the DAI scores. The protective effects of CBR extracts may be attributed to a significant reduction in the levels of iNOS and COX-2 proteins and TNF-*α* and IL-6 production. The inhibitory activity of the CBR extract against these inflammatory mediators may be due to the regulation of the NF-*κ*B and MAPK signaling pathways. These results suggest that the CBR extract might be a potent anti-inflammatory candidate for preventing IBDs.

## Figures and Tables

**Table 1:** Disease activity index (DAI) scoring. The sum of scores for the DAI category.

Score	Symptom		
	Weight loss	Stool consistency	Bleeding
0	no	Normal stools	Normal
1	1%–5%		
2	6%–10%	Loose stools	Slight bleeding
3	11%–20%		
4	~20%	Diarrhea	Gross bleeding
